# The Role of Grunt Calls in the Social Dominance Hierarchy of the White-Lipped Peccary (Mammalia, Tayassuidae)

**DOI:** 10.1371/journal.pone.0158665

**Published:** 2016-07-13

**Authors:** Selene S. C. Nogueira, Christini B. Caselli, Thaise S. O. Costa, Leiliany N. Moura, Sérgio L. G. Nogueira-Filho

**Affiliations:** 1 Departamento de Ciências Biológicas, Universidade Estadual de Santa Cruz, Rod. Jorge Amado, km 16, Ilhéus, BA, 45662–900, Brazil; 2 Departamento de Ciências Agrárias e Ambientais, Universidade Estadual de Santa Cruz, Rod. Jorge Amado, km 16, Ilhéus, BA, 45662–900, Brazil; University of Pisa, ITALY

## Abstract

Grunt-like calls are present in the vocal repertoire of many group-living mammals and seem to facilitate social interactions between lower and higher-ranking members. The white-lipped peccary (*Tayassu pecari*) lives in stable hierarchical mixed-sex groups and like non-human primates, usually emits grunt-like calls following aggressive interactions, mainly during feeding contexts. We investigated the possible functions of peccaries’ grunt-like calls and their relationship to the individuals’ social rank, identity, and sexual dimorphism. We observed that low-ranking individuals emitted grunt-like calls more often than high-ranking ones, and that the alpha male never emitted this vocalization. Moreover, the mean minimum frequency of grunt-like calls decreased as the peccary’s rank increased. The findings revealed differences among individual grunts, but the low accuracy of cross-validation (16%) suggests that individual recognition in peccaries may be less important than an honest signal of individual social status. In addition, the absence of differences in the acoustic parameters of grunt-like calls between males and females points to the lack of sexual dimorphism in this species. We verified that after hearing grunt calls, dominant opponents were more likely to cease attacking a victim, or at least delay the continuation of conflict, probably decreasing the severity of agonistic interactions. Our findings are particularly important to improve the current understanding of the role of grunt-like calls in herd-living mammals with linear dominant hierarchies, and strongly suggest that they are involved in the maintenance of herd social stability and cohesion.

## Introduction

Acoustic signals play an important role in social interactions among mammals [1–4] and birds [5–6]. In mammals, vocalisations seem to contribute to social interactions such as partner and offspring recognition [7–11], to promote group cohesion [1,12–14], and to facilitate social interactions [15–18]. In some species, calls may play more than one functional purpose and encode information on sex, age and social status. For example, the hyena’s (*Crocuta crocuta*) giggle, often referred as the hyena’s laugh, encodes information about the animal’s identity, social status and age [17]. After hearing this call from one individual, hyenas’ clan-mates can decide whether to join the giggler or not depending on caller identity [17]. In white rhinoceros (*Ceratotherium cottoni*), the panting call is used as a greeting and for sex and age recognition [18–20]. During territorial encounters, rhino males are more likely to react more strongly to panting from a subordinate male, possibly to confirm its social status [21].

Grunt-like calls are present in the vocal repertoire of many group-living mammals and seem to facilitate social interaction, especially between lower and higher-ranking group members [15], [16], [22], [23]. In chimpanzees (*Pan troglodytes*), which live in a fission-fusion society with linear hierarchy, for instance, the pant-grunt vocalization is typically a greeting signal [24]. This kind of call is emitted by lower-ranking individuals towards higher-ranking males or females [25]. In addition, pant-grunts were reported to play a role in reconciliation after aggressive encounters [24], [26] or to express fear [26], [27], and as a submissive-signal [22], [27]. In rhesus macaque (*Macaca mulatta*) and baboons (*Papio cynocephalus ursinus*), dominant females usually emit quiet grunts to communicate their intent to behave peacefully [15], [23], [28]. In the white-lipped peccary (*Tayassu pecari*), a group-living mammal, grunts are also present [29] and may be used to facilitate social interactions.

**Fig 1:**
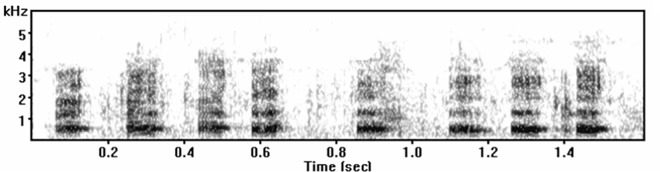
Grunt call emitted by a white-lipped peccary (*Tayassu pecari*).

The white-lipped peccary is an ungulate that lives in dense Neotropical forests [29] in mixed-sex and stable herds numbering in the hundreds [30]. These herds are characterised by high cohesiveness, even during seasons of food-shortage [30], [31]. In captivity, the species shows a strict dominance hierarchy in which both males and females can reach the highest dominance rank [32], [33]. Although little is known about that peccary’s vocal repertoire [29], the white-lipped grunt is their most frequent vocalisation; it is a loud, conspicuous, fast-rising and noisy sound emission used for short-distance communication [29], [32], [33], [34] (Fig 1). The grunts are usually emitted following aggressive interactions during feeding time in both captive and free-ranging animals [29], [32], [33], [34]. Because of the context in which this vocalization is used, it has the potential to mediate social conflicts by preventing or mitigating aggression, contributing to the stability of that peccary group.

Our aim, therefore, was to investigate the possible functions of peccary’s grunts and their relationship to an individual’s social rank, identity and sexual dimorphism. If the grunt-like calls are used to mitigate conflicts by signaling the caller’s social status, as observed in non-human primates [22], we expect that lower-ranking individuals will emit grunts more often than dominant ones to indicate their submissive status. Furthermore, the acoustic structure of calls used to mediate agonistic interactions may provide cues about the sender’s motivational state [35], [36]. For instance, aggressive individuals usually produce low-frequency sounds with a broad frequency band, while frightened submissive individuals produce sounds with higher frequency and narrow frequency band [35]. Together with low-frequency sounds, spectral noisy vocalizations, which reflect patterns of deterministic chaos in laryngeal vibration [37], can also provide cues about the individuals’ dominance rank [38] and about the severity of excitement or aggression [36], [39]. Thus, in our study we also expect higher-ranking individuals to demonstrate willingness to counter-attack in a stronger manner than lower-ranking individuals, using longer, noisier and lower-frequency calls [35], [36]. In addition, we do not expect to find individual differences in grunts, because individual recognition is not necessary for conflict avoidance [16], [22]. Finally, as females seem to be valuable in peccary society, as would be suggested by the fact that they usually occupy higher ranks after farrowing (giving birth to a litter) [32], we would expect to find acoustic differences in grunts between sexes to allow female recognition, given that there are no obvious visual cues that permit sexual identification.

## Methods

### Ethics Statement

This work followed the principles of laboratory animal care (NIH publication No. 86–23, revised 1985) and was approved by the Committee on Animal Research and Ethics of the State University of Santa Cruz, under protocol no. 010/11.

### Study site and animals

We conducted an observational study with 19 adult white-lipped peccaries (9 males, 10 females) at the Applied Ethology Laboratory of the Universidade Estadual de Santa Cruz, Bahia, Brazil (14°47’39.8”S, 39°10’27.7”W). The animals (Table 1) were all adults (M_weight_ = 39.3 SD = 6.0) from the same family herd (N = 19) strongly related, born and raised in captivity. Because the animals came from another captive breeding center, we could not determine their exact age (range: 3–8 years old). The animals were identified with plastic ear tags of different shapes and housed in a 940m² paddock surrounded by a 1.5 m high wire fence. The paddock had a dirt floor with the presence of medium-sized and tall trees and bushes, which provided natural shade. Food was provided twice a day (at 8:30 am and 4:00 pm) and water was available *ad libitum* in a water trough.

**Table 1:** Mean values (± standard deviation) of the acoustic parameters for grunts emitted by white-lipped peccaries according to the ranking position. Lower numbers correspond to higher positions in the dominance hierarchy. All measurements were taken from 50 samples from each individual. The percentage of correct cross-classified grunt calls for each individual based on the conventional (DFA) and permuted discriminant analysis (pDFA) is also presented, based on 37 calls for each individual.

Animal	Ranking position	Sex	Weight	Duration (s)	Peak frequency centre (Hz)	Mean minimum frequency (Hz)	Mean bandwidth (Hz)	HNR centre (dB)	% Corrected cross-classified (DFA)	% Corrected cross-classified (pDFA)
Losango	1	M	33	-	-	-	-	-	-	-
Alfa	2	F	30.4	0.07 ± 0.02	568.0 ± 196.7	353.1 ± 78.3	1451.3 ± 599.3	32.1 ± 10.6	10	12.5
Bola	3	F	42	0.10 ± 0.07	694.4 ± 346.1	366.0 ± 83.3	1331.2 ± 531.6	22.4 ± 10.4	32	21.7
Coração	4	F	43.8	0.07 ± 0.02	846.4 ± 590.6	392.2 ± 111.7	1987.0 ± 588.9	30.9 ± 13.3	22	8.8
Cruz	5	F	42.4	0.17 ± 0.12	687.4 ± 253.1	368.9 ± 115.0	1364.6 ± 616.9	33.0 ± 10.0	48	49.4
Pente	6	F	45	0.18 ± 0.17	686.0 ± 308.1	344.0 ± 129.4	1425.3 ± 424.5	28.2 ± 13.0	10	6.5
Nuvem	7	F	39.4	0.12 ± 0.07	1183.6 ± 871.2	399.0 ± 262.1	1993.0 ± 964.4	34.4 ± 13.9	8	5.3
S	8	M	40.6	0.10 ± 0.04	1006.0 ± 521.8	401.4 ± 100.6	2484.8 ± 1183.5	28.5 ± 12.4	29	24.9
Furinho	9	M	52	0.07 ± 0.03	671.8 ± 348.7	372.1 ± 112.8	1479.2 ± 635.0	34.0 ± 12.5	24	28.0
Radioativo	10	M	37.4	0.08 ± 0.02	703.0 ± 440.5	332.6 ± 103.5	1580.9 ± 568.9	29.7 ± 10.8	13	10.6
Brinco	11	F	47.4	0.14 ± 0.12	898.2 ± 376.1	377.9 ± 140.5	1920.2 ± 690.6	24.2 ± 12.0	9	4.7
Garfo	12	M	38.4	0.08 ± 0.02	687.2 ± 284.6	413.3 ± 124.4	1274.9 ± 534.0	32.9 ± 9.9	3	5.0
Árvore	13	F	43.4	0.07 ± 0.02	769.8 ± 505.2	413.6 ± 102.6	1346.9 ± 544.0	23.0 ± 13.3	15	18.5
Serrote	14	M	36.4	0.08 ± 0.02	898.4 ± 457.8	424.8 ± 124.3	1700.8 ± 665.2	33.7 ± 13.0	0	0.0
Retângulo	15	M	34.2	0.07 ± 0.02	759.4 ± 375.3	363.9 ± 106.2	1674.9 ± 528.1	31.3 ± 13.0	41	44.8
T	16	M	29.8	0.11 ± 0,06	771.2 ± 406.9	404.4 ± 137.3	2107.8 ± 779.8	23.1 ± 13.3	33	24.5
Ameba	17	F	44.2	0.09 ± 0.05	658.6 ± 369.0	351.5 ± 130.4	1770.1 ± 800.3	26.7 ± 13.9	2	0.0
L	18	M	38	0.08 ± 0.02	930.2 ± 573.8	435.4 ± 114.4	1633.8 ± 717.8	25.5 ± 13.1	5	5.5
Z	19	F	29.8	0.08 ± 0.02	963.2 ± 511.3	445.4 ± 90.9	1826.8 ± 607.4	21.6 ± 14.6	22	23.5

### Data collection

We conducted the observations during feeding times in the morning or afternoon, because most of the conflicts in peccaries occur during competition for food, both in captivity and in the wild [29], [32], [33]. During this time and concomitantly to the grunt recording (see below), one auxiliary person videotaped (JVCGZ-HD500, Tokyo, Japan) the entire group for further analyses of all occurrences [40] of agonistic interactions. Inter-observer reliability was not calculated because only one observer (Costa, TSO) analysed the social interactions. Agonistic interactions, such as threats, chases and attacks with direct physical contact, as well as all submissive behavioural patterns, such as escape and head lowering to show submission, which occurred together with grunts, were analysed. Only the conflicts that clearly allowed scoring the individuals as either winner or loser after each aggressive interaction were submitted to further analyses (see details in the section Social dominance analysis*)*.

All grunt calls were recorded in WAV format, mono mode, using a Sennheiser ME-67 directional microphone (Sennheiser Communications A/S, Solrod Strand, Denmark) and a Marantz PMD-671 (D&M Holdings, Inc, Mahwah, NJ) digital recorder at 48 kHz sampling rate and 16-bit resolution. For each grunt, the observer noted the signaller and receiver identity; the behavioural patterns involved, and recorded the escalation of aggression after the vocalization, by recording occurrences of fight or non-fight patterns. During white-lipped peccary conflicts, the aggressor usually heads towards the opponent and the victim walks backwards or runs away from the aggressor. Thus, the observer used these behavioural cues to infer the identity of the receiver of these vocalisations.

Each observation/recording session lasted 1 h per day, totalling 60 h of data collection over four months. Because all the animals were fully habituated to human observers, we were able to collect and record all behavioural data and vocal emissions at close range–from 2.0 to 5.0 m away from the animals. The majority of agonistic interactions occurred close to the observation point, making it easy to identify the interacting individuals and record their calls.

### Social dominance analysis

We analysed the dominant-subordinate relationships using the approach described by Lehner [41]. Each individual was scored as either the winner or loser after each aggressive interaction. Following the procedures described by Nogueira-Filho *et al*. [33], the loser was defined as the animal that moved away or fled from the winner or showed submissive displays, such as lowering the head or the anterior part of the body. To test for linearity of hierarchy, we calculated Landau’s corrected linearity index (h’), adjusted for unknown relationships using SOCPROG 2.4 [42]. This index ranges from 0 (nonlinear hierarchy) to 1 (perfectly linear hierarchy). The index h’ takes into account the existence of unknown relationships [43] and the statistical significance of h’ is provided by a resampling procedure using 10,000 randomisations [42]. SOCPROG also provided a rank order for each individual in both periods through the I & SI method [44]. The I & SI method aims to determine a rank order most consistent with a linear hierarchy by first minimising the number of inconsistencies ‘I’ and, subsequently, minimising the total strength of the inconsistencies ‘SI’, subject to the condition that ‘I’ does not increase [45]. The I & SI method minimises the number of inconsistencies by minimising the sum of the rank differences between individuals whose ranks were inconsistent [44]. In addition, we calculated the directional consistency index (DCI) [46]. The resulting values range from zero (complete bidirectional exchange of submissive gestures) to one (complete unidirectionality).

### Acoustic analyses

Grunts are mainly produced in bouts of concatenated vocal units or elements, which generate uninterrupted spectrographic tracings that are separated by silent intervals (Fig 1). From among the 4,021 elements recorded, we chose 1,849 samples with higher signal quality, less background noise and no overlap among calls from different individuals. From these samples we randomly chose 50 samples of grunts [see S1 Dataset] from each individual (N = 19) for acoustic measurement. Since one of the animals did not emit grunts (see Results), we performed the acoustic analyses using 900 samples. We also selected 90 bouts of grunts, five from each animal, to characterise total bout duration and the mean number of elements emitted in each bout. For the acoustic analysis, however, we focused on the acoustic features that could be measured from a single element, because each element can vary and provide information on an individual’s identity [47], [48].

We conducted the acoustic analyses using the automatic parameter measurement tool of Avisoft-SASLab Pro 5.2.05 software (R. Specht, Berlin, Germany), with the following settings: two thresholds of -9 and -10 dB, 50 ms hold time, 1,024 FFT size, 87.5% overlap, Hamming window, 2.7 ms time resolution and 47 Hz frequency resolution. The use of the automatic parameter measurement tool is important to minimise the influence of subjectivity in measuring acoustic parameters. Following Caselli [14], before conducting automated measurements, we normalised each call to the same maximum amplitude (-1 dB) and filtered all sounds above 30 kHz and below 8 kHz to standardise calls across recordings made from variable distances from the source and to eliminate background sounds outside the frequency range of peccary vocalisations. For each element, we measured its duration (s) and six additional acoustic structural parameters: peak frequency (Hz), minimum frequency (Hz), maximum frequency (Hz), bandwidth (Hz), harmonic-to-noise ratio (dB), and entropy. Because the grunts are characterized by low modulation in frequency, we measured these last six parameters at the centre of each call and throughout all call durations. The minimum and maximum frequencies correspond to the least and greatest frequency of each element, considering the amplitude threshold used for automatic measurements, and the bandwidth corresponds to the difference between these values. The harmonic-to-noise ratio (HNR) parameter is related to the amount of noise within a signal and decreases as the amount of noise increases [49], [50]. We measured this parameter only at the centre of the call. The entropy quantifies the pureness of sounds. Theoretically, this parameter is zero for pure-tone signals and one for random noise [51]. We chose to measure these parameters because in the noise pattern of the atonal grunts it is not possible to detect the fundamental frequency (F0) and its harmonics. We then focused on other acoustic parameters also indicated to contribute to discriminating an individual, such as duration, minimal frequency and frequency range or bandwidth [17], [39], [52]. Formant-like spectral features (vocal tract resonances) [53] can be observed in peccaries’ grunts, but we did not measure these formants due to the lack of appropriate knowledge on peccaries’ vocal tract morphology. Thus, we included the measurement of peak frequency, which reflects the frequency of the formant with most energy [53]. HNR can potentially provide information about the emotional state of individuals [36] and also be related to the perceptual characteristics of acoustic signals [54]. As this study was conducted in a context of great arousal and we expected that individuals would differ in their arousal state due to their rank and the effect they might have on the receiver, we also expected that these parameters would be informative.

### Data Analysis

To verify the potential of a grunt to prevent continued aggression within white-lipped peccary group members, we performed a goodness-of-fit test to compare whether the outcome after grunt emission was an end to the conflict or continuation of aggression. We also measured the time taken between grunt emission and cessation of the conflict or continuation of aggression. To evaluate if a grunt was used to mitigate conflicts by signaling individuals’ social dominance status [15], [22], whether by the number of emissions and/or distinct acoustic structure according to dominance rank, we used Spearman rank correlations for individuals’ rank and the following parameters: number of grunts emitted, element acoustic structure–mean bandwidth (Hz), mean minimum frequency (Hz), and mean HNR (dB).

To test whether we could assign grunts to individuals or sex, we performed conventional and permuted discriminant function analyses. Permuted discriminant function analysis (pDFA) is suitable for nested and non-independent data and was employed to estimate the overall significance of discriminability, since conventional DFA does not allow for a valid estimation in these cases [55]. We thus performed pDFA using nested designs to estimate the significance of discriminability between sexes (controlling for individual’s identity) and among individuals (including sexes as restriction factors).

Before conducting the discriminant analyses, we applied the Yeo-Johnson power transformation [56] to the predictor variables for reducing skewness and to approximate normality. For this, we used the *preprocess* function from the R package “caret” version 6.0–64 [57]. To avoid the influence of extreme data we also checked for outliers using the function *mvoutlier*.*CoDa* to compute multivariate outliers implemented in the package “mvoutlier” version 2.0.6 [58]. Because it is likely that some of the measured acoustical parameters are correlated, we checked for collinearity in variables using the variance inflation factor (VIF). A VIF greater than 2 can be an indicator that the model has moderate or high collinearity [59], and thus we excluded the variables with VIF values greater than 2. We used a stepwise procedure, first detecting a pair of variables with maximum linear correlation and excluding the one with greater VIF. The procedure was repeated until no variable with VIF greater than two remained. This procedure was conducted using the “usdm” package version 1.1–15 [60]. We then performed the discriminant analyses with five of the 12 original measured acoustic parameters (duration, peak frequency center, HNR center, mean minimum frequency, and mean bandwidth), which is in accordance with the recommended number of variables used to derive the discriminant functions: less than the number of objects in the smallest level of variable to be tested for discriminability [61].

From the total 900 samples (50 for each individual), 138 were identified as outliers and removed from the analyses. We thus conducted the discriminant analyses with 762 samples. To construct a balanced conventional DFA model for comparing individuals, we used 37 samples per subject (which is seven times greater than the number of variables). To construct the permuted DFA to estimate the significance of discriminability between callers, we first randomly selected 37 samples per subject and then repeated this procedure 100 times to calculate the average percentage of correct classifications for the original data set. To compare the classifications of calls for the original data set with that expected by chance, we then randomly selected 37 calls per subject and randomly assigned these calls to one of the 18 individuals. We repeated this procedure 100 times to derive the average percent correctly classified calls of the permuted data. To construct balanced models for the pDFA comparing sexes, we used the same approach. We first randomly selected 36 samples of 8 randomly selected individuals per sex and then repeated this procedure 100 times. We then randomly selected 36 calls from eight individuals and randomly assigned these calls to one of the sexes. We repeated this procedure 100 times to derive the average percentage of correctly classified calls of the permuted data. The number of samples arranged by sex (36 samples times eight individuals per iteration) was seven times greater than the number of variables. The samples not used to construct the discriminant models were used to cross-validate the data. When the pDFA model was significant, we also presented the results of cross-validation analysis based on the conventional DFA and verified it using a binomial test. The pDFA was conducted in R (version 3.2.3; R Core Team 2015) using a function written by R. Mundry based on the function lda of the R package “MASS” version 7.3–45 [62].

## Results

From the 1,880 agonistic interactions observed among all dyads of the group, we obtained a linear dominance rank order (linearity index *h*’ = 0.9; *P* < 0.0001) Table 1), which included both males and females without circular relationships. Moreover, the directional consistency index (DCI = 0.9) showed an almost complete unidirectionality of submissive gestures.

The dominant male was the only individual that did not emit grunts (Table 1). The lower the animal rank, the higher the number of grunts emitted (r_s_ = 0.54, N = 19, *P* < 0.01). Moreover, while the mean minimum frequency of grunts decreased as the peccary’s rank increased (r_s_ = 0.52, N = 18, *P* < 0.05), the other two acoustic parameters were not correlated with animal rank (mean HNR: r_s_ = -0.24, N = 18, *P* = 0.34; mean bandwidth: r_s_ = 0.24, N = 18, *P* = 0.33). We verified that grunt production often resulted in the end of conflict (N = 400) instead of aggression continuation (N = 13) (Chi square = 362.64, df = 1, *P* < 0.0001). Moreover, the end of conflict in response to a grunt occurred on average (±SD) of 1.3±0.6s after the grunt (N = 400), while subsequent aggression occurred on average (±SD) of 6.8±5.1s following the grunt (N = 13).

**Fig 2:**
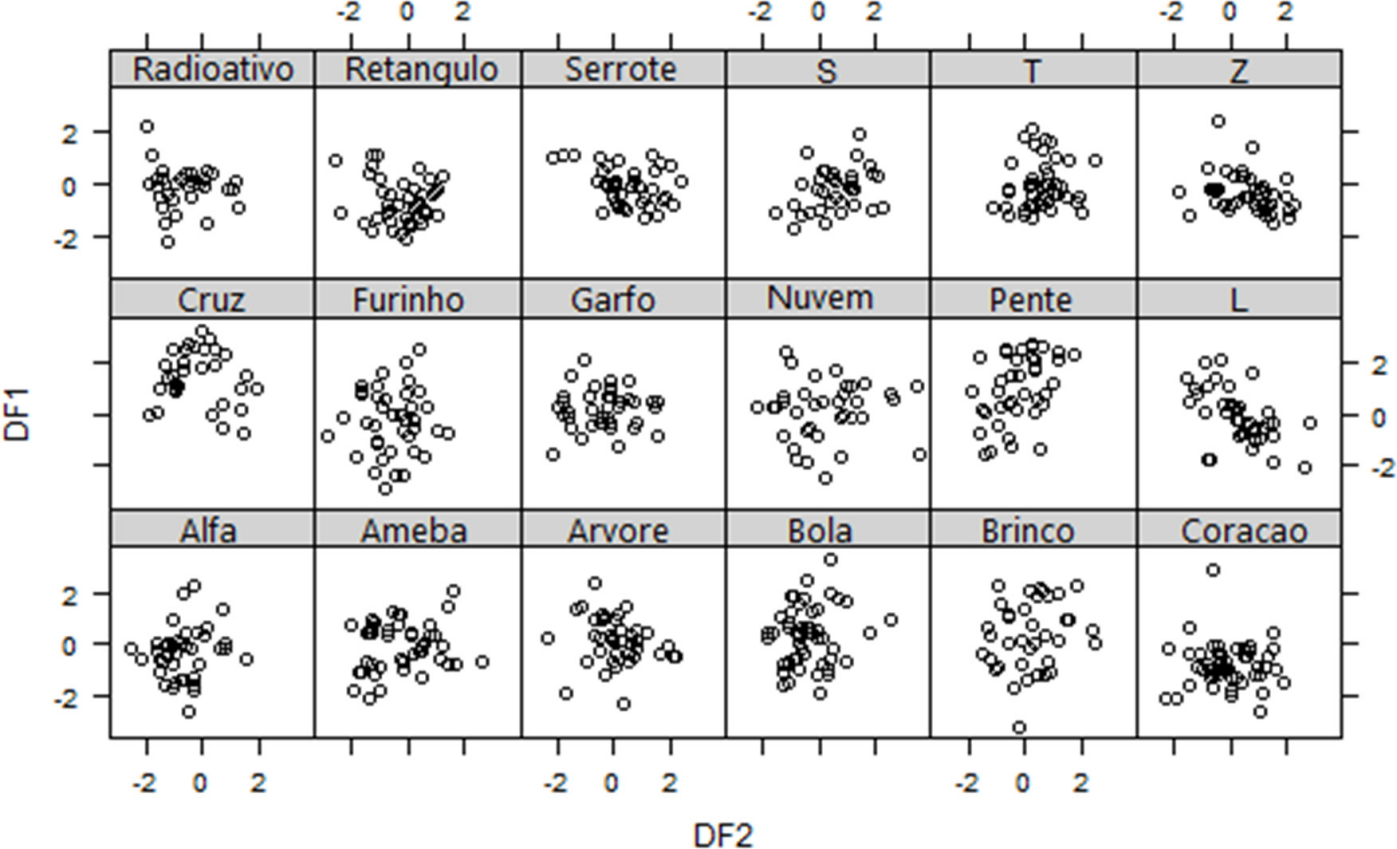
Distribution of discriminant scores for each individual along the two canonical discriminant functions.

Grunts were emitted as solo units (elements) or sequences of 2 to 20 elements (mode = 3, N of sequences = 90), with a mean duration of 1.63 ± 1.34 sec (mean ± SD, N of sequences = 90). The conventional DFA differentiated the grunts among individuals (Wilks Lambda = 0.52, *P* < 0.0001, N = 666). The first two discriminant functions (DF) explained 61% of the variation in the acoustic structure of individuals’ grunts (Table 2). The pulse duration (s) showed the highest loading for DF1, while the peak frequency centre (Hz) showed the highest loading for DF2 (Table 2). The greatest source of signal discrimination came from the peak frequency, which showed the highest load of all variables. Cross-validation analysis based on conventional DFA correctly assigned the grunts to each sender with a mean accuracy of 18.5%, which was higher than that expected by chance (5.6%; Binomial test: *P* < 0.001). The permuted DFA (pDFA) confirmed that individuals vary in the acoustic structure of their grunts (*P* < 0.001). Cross-validation analysis correctly assigned the grunts to each sender with a mean accuracy of 16% (based on 100 random selections of the original data). Although the percentage of correct classification was very low, it was greater than the 6% expected by chance (based on 1000 permutations of the original data set). The cross-validation revealed a great variation in the percentage of correct classification across callers, ranging from 0% to 49% (Table 1; Fig 2). The pDFA revealed that there was no difference between the acoustic features of male and female grunts. Although the cross-validation correctly assigned the grunts to males and females with a mean accuracy of 53.72% (based on 100 random selections of the original data), the original data set does not classify better than the permuted data (average percentage of correct classifications for permuted data set = 53.36; *P* = 0.50).

**Table 2:** Coefficients of the two main discriminant functions (DF) indicating the relative contribution of each acoustic parameter (variable) for discriminating between individuals’ grunt calls. The values in bold indicate the parameters with higher loading for the discriminant functions (DF1 and DF2).

Acoustic parameters	DF1	DF2
Peak frequency centre (Hz)	0.2640	**0.6190**
Duration (s)	**0.1260**	0.0390
Mean bandwidth (Hz)	-0.0100	0.0060
Mean minimum frequency (Hz)	-0.0001	0.0010
HNR (dB)	-0.0020	-0.0510
Cumulative percentage of explained variance	38	61

HNR, harmonic-to-noise ratio

## Discussion

Our study supported our prediction that grunt-like calls play a role in white-lipped peccary social interactions by preventing or mitigating aggression. Low-ranking callers emitted grunts more often than higher-ranking ones, whereas the alpha male never emitted this vocalization. We verified that after hearing grunt emissions, dominant opponents ceased the conflict or at least delayed the continuation of aggression for some seconds, possibly reducing the severity of agonistic interaction. The DFA indicated that grunts were individually distinctive; however, a low accuracy of assigning grunts to individuals was detected. These results seems to point to at least two scenarios: (i) individual recognition is not the main function of grunts, perhaps because it is not necessary for peccary social organization, or (ii) the acoustic parameters used in the present study did not allow acoustic cues to the callers’ identity to be detected. Moreover, contrary to our expectations, there was no sexual dimorphism in the acoustic parameters of grunts in this species.

Top-ranking white-lipped peccaries emitted grunts with a lower mean minimum frequency. The higher pitched grunts of subordinate peccaries may be influenced by social experience, and may reflect affective state and levels of excitement or aggression [35], [36]. The observed correlation between individuals’ rank and mean minimum frequency among animals could also be explained by individuals’ age and consequent weight, instead of social rank. The animals' body size is likely to affect the spectral amplitude distribution, such as peak frequency, which may have lower values in larger animals [63]. In southern white rhinos, for instance, pants showed differences between sex, depending on the age class and social status of the signaller [21]. As all peccaries in this study were adults and their weight was similar (Table 1), we did not expect to find any influence of age on individuals’ rank and minimum frequency. Here the correlation between animals’ rank and mean minimum frequency may be more reasonably explained by the affective state of the individuals [35]. Mathevon et al. [17], studying *Crocuta crocuta*, in accordance with Morton rule [35], report that aggressive individuals usually produce lower frequency sounds with a broad frequency band, while frightened submissive individuals produce sounds of higher frequency and a narrow frequency band.

Once the hierarchy is established, the alpha male is not threatened by other group members [32], [33, 64], and this could explain the fact that the alpha male did not emit grunts during our study. In contrast, lower-ranking peccaries needed to signal their rank in a more conspicuous way, which allowed dominants to modulate their response, avoiding unnecessary levels of aggression. By signalling its submission status, the subordinate peccary can provide a ritualized way to avoid or prevent continuation of aggression. Grunts in peccaries show some similarities with non-human primates. The pant-grunt, for instance, which is primarily considered as greeting signal in chimpanzees [24], is also related to dominance-submission relationships [65], because subordinate individuals usually address pant-grunts to dominant males or females [22], [24], [25]. Sakamaki [22] reported that pant-grunt vocalization in chimpanzees was present in 80% of aggression encounters; similarly, peccaries usually emit frequent grunts in social conflicts during feeding [29], [32], [33]. In chimpanzees, it was also observed that pant-grunts together with agonistic interactions participate to form a single hierarchy of social status allowing males to be ranked in distinct social levels [66]. In savannah baboons, grunts are also related to the social status of individuals involved [16]. This vocalization, however, occurs in a different situation from the peccary’s grunts. While grunts in peccaries are emitted by subordinates, the dominant female in baboons uses a grunt call to the subordinate that then allows the former dominant to approach less aggressively, as a reconciliatory interaction [16]. The authors suggest that the grunt can reduce victims’ anxiety and can also facilitate interaction between adversaries, despite not changing their social relationships [67]. Therefore, our findings suggest that grunts play a role in the social dominance of peccaries, similarly to what occurs in chimpanzees [22], [66]. Moreover, rather than reducing aggression *per se*, as happens among baboons [16], peccary’s grunts may help to maintain (at least in captivity) the strict dominance hierarchy among group individuals.

The low accuracy of grunt identification suggests that caller recognition in peccaries may be less important than its function as a strong sign of individual social status. In baboons, for instance, the authors suggest that reconciliatory grunts may indicate the low probability of aggression occurrence, and victims possibly learn by experience or observation [16]. Cheney and Seyfarth [16], suggest that in baboons, grunts can alter the recipient’s behaviour and increase the probability that dominant females will be able to interact with victims (subordinate). Thus, our results indicate that the most important function of grunts in peccary society is to indicate submission and refusal to be supplanted by dominants, as observed in baboons. Furthermore, white-lipped peccaries live in herds which number up to 300 individuals [30] and this makes it more difficult to assess any kind of acoustic parameters associated with identity. Bergman [68], comparing individual recognition among males in geladas (*Theropithecus gelada*) and baboons (*Papio ursinus*), hypothesized that to track individual social information of many individuals (baboons: 2–15 and geladas: over 30 in band and 100 in herd) is cognitively challenging for geladas, which we suggest is also true for peccaries.

Our prediction of sexual differences in grunt vocalization has to be rejected, in line with previous studies, which did not find any dimorphic differences between males and females of white-lipped peccary and suggest that peccary groups compete equally for limited resources and establish a unique social dominance hierarchy [32], [33]. In spotted hyenas (*Crocuta crocuta*), for instance, the giggle calls do not show reliable differences between male and females [17]. Therefore, other acoustic parameters such as formant-like spectral features (vocal tract resonances) may provide information on sex differences and animals’ identity. However, in the present study we could not measure the peccary’s formants due to the lack of appropriate knowledge about peccaries’ vocal tract morphology. In addition, chemical communication could also support some sexual recognition in white-lipped peccary [29], but further studies need to be carried out to answer the dimorphism question as reported for other species [69], [70].

Our findings are particularly important to improve the current understanding of the role of grunt-like calls in herd-living mammals with linear dominant hierarchies, which strongly suggests that they are involved in the maintenance of herd social stability and cohesion. Although further studies are required to improve our understanding of the acoustic function and structure of grunts and other white-lipped peccary vocalizations, this work is the first step in improving our knowledge of their acoustic repertoire.

## Supporting Information

S1 Dataset Acoustic parameters of white-lipped peccaries’ grunt calls. (XLS)
